# Enhanced anti-metastatic and anti-tumorigenic efficacy of Berbamine loaded lipid nanoparticles *in vivo*

**DOI:** 10.1038/s41598-017-05296-y

**Published:** 2017-07-19

**Authors:** Priyambada Parhi, Sujit Suklabaidya, Sanjeeb Kumar Sahoo

**Affiliations:** 1 Institute of Life Sciences, Nalco Square, Chandrasekharpur, Bhubaneswar, India; 2 Manipal University, Karnataka, India

## Abstract

Research on metastasis is gaining momentum for effective cancer management. Berbamine (BBM) has the potency to act as a therapeutic in multiple cancers and cancer metastasis. However, the major limitation of the compound includes poor bioavailability at the tumor site due to short plasma half-life. Here, our major objective involved development of lipid based nanoparticles (NPs) loaded with BBM with an aim to circumvent the above problem. Moreover its, therapeutic potentiality was evaluated through various *in vitro* cellular studies and *in vivo* melanoma primary and experimental lung metastatic tumor model in C57BL/6 mice. Results of different cellular experiments demonstrated enhanced therapeutic efficacy of BBM-NPs in inhibiting metastasis, cell proliferation and growth as compared to native BBM in highly metastatic cancer cell lines. Further, *in vivo* results demonstrated suppression of primary B16F10 melanoma tumor growth in C57BL/6 mice model treated with BBM-NPs than that of native BBM. Importantly, a moderately cytotoxic dose of BBM-NPs was able to significantly suppress the incidence of B16F10 cells lung metastasis *in vivo*. Results indicated development of an effective approach for aggressive metastatic cancer.

## Introduction

Despite new advancement of different therapeutics for treatment of cancer, till date metastasis is regarded as one of the most formidable bottleneck for effective cancer management. In majority of the cancer patients, distant metastases are known to be the primary cause of death^1, 2^. Tumor metastasis consists of series of distinct biological processes which includes invasion of tumor cells from the primary tumor, intravasation of those cells either to lymphatics or the blood stream, survival of the cells in circulation, arrest at a new site, extravasation and growth in different microenvironment^3–7^. In this metastasis cascade, multiple proteins such as adhesion molecules, extra cellular matrix degrading enzymes, cytoskeleton modulators etc. play a major role^8, 9^. Thus, a strategy that can target different factors, which instigate the migration of cancer cells from the original location to distant sites to form new tumors, may pave a new way for effective cancer treatment.

Recently, identification of anticancer drugs from natural products including plants, microorganisms has been the hub of research worldwide^10, 11^. In this context, Chinese herbal medicines which include curcumin, berberine, quercetin etc. have been well documented for the treatment of metastatic cancer in recent years due to their wide range of biological activities with reduced toxicity over chemotherapeutic regimes^12–14^. In this regard, Berbamine (BBM), a bisbenzylisoquinoline alkaloid extracted from a plant *Berbaris amurensis* has shown its potency against multitude of diseases including cancer^15^. Recently, the potent anticancer activity of BBM in diverse cancer type including breast, hepatoma, leukemia, lung cancer etc. has received great attention in cancer research. BBM shows its anticancer activity by induction of apoptosis, cell cycle arrest^16^ and reversing multidrug resistance^17^. Recently, it is being reported that, BBM can effectively inhibit tumor metastasis by suppressing cell proliferation, migration and invasion in highly metastatic breast cancer cells under *in vitro* condition^18^, thus indicating the potentiality of this molecule as next generation anti-metastatic agent.

Usually, chemotherapeutics/herbal extracts have very short plasma half-life leading to poor bioavailability at the tumor site after systemic administration. Thus, delivering therapeutic dose of BBM in sustained fashion at tumor site is a prerequisite for clinical implementation of this potent anticancer drug. In this context, nanotechnology has paved the way for new discoveries of drug delivery vehicles by improving the clinical therapeutic efficacy of the drugs by increasing the amount of drug deposited in the tumor tissues while decreasing its accumulation in healthy tissues, for better cancer treatment^19^. Among various nanocarriers, lipid based NPs are considered to be one of the most promising nano-drug delivery vehicle due to their small particle size, capability to cross different biological barriers and potency to enhance the accumulation of drugs at the target site to achieve efficient delivery of chemotherapeutic drugs^20, 21^. We have shown in our previous work that lipid based NPs can be used as a nanotheranostic approach for simultaneous diagnostics and therapeutics in an effective manner than that of native drug for improvement of breast cancer therapy^22^. Our group has also shown higher bioavailability of curcumin by using lipid based NPs in an animal model^23^. Furthermore, NPs less than 100 nm in size are able to take the advantage of pathophysiological features in tumors and extravasate through leaky vasculature of tumor tissues and accumulate within the extracellular matrix due to impaired lymphatic drainage, collectively referred as enhance permeability and retention effect^24^.

Thus, the present investigation aims on formulating BBM loaded NPs (BBM-NPs) for the treatment of metastatic cancers in both *in vitro* and *in vivo* tumor model. The dynamic approach of the current work is to evaluate the anticancer and anti-metastatic efficacy of BBM-NPs on tumorigenesis and metastasis of highly metastatic MDA-MB-231 breast cancer, A549 lung cancer and B16F10 mouse melanoma cell lines by performing various *in vitro* cellular studies. Further, the therapeutic evaluation of BBM-NPs on primary tumor was evaluated by melanoma (B16F10) subcutaneous tumor model and effect on metastasis was evaluated through hematogenous metastasis of melanoma cells to lung. Results demonstrated that BBM-NPs can effectively inhibit the primary tumor growth and lung metastasis in melanoma tumor model using C57BL/6 mice. Thus, the above study signifies that, BBM-NPs can be a better candidate for the treatment of metastatic cancer.

## Results

### Physico-chemical characterization of NPs

**Figure 1:**
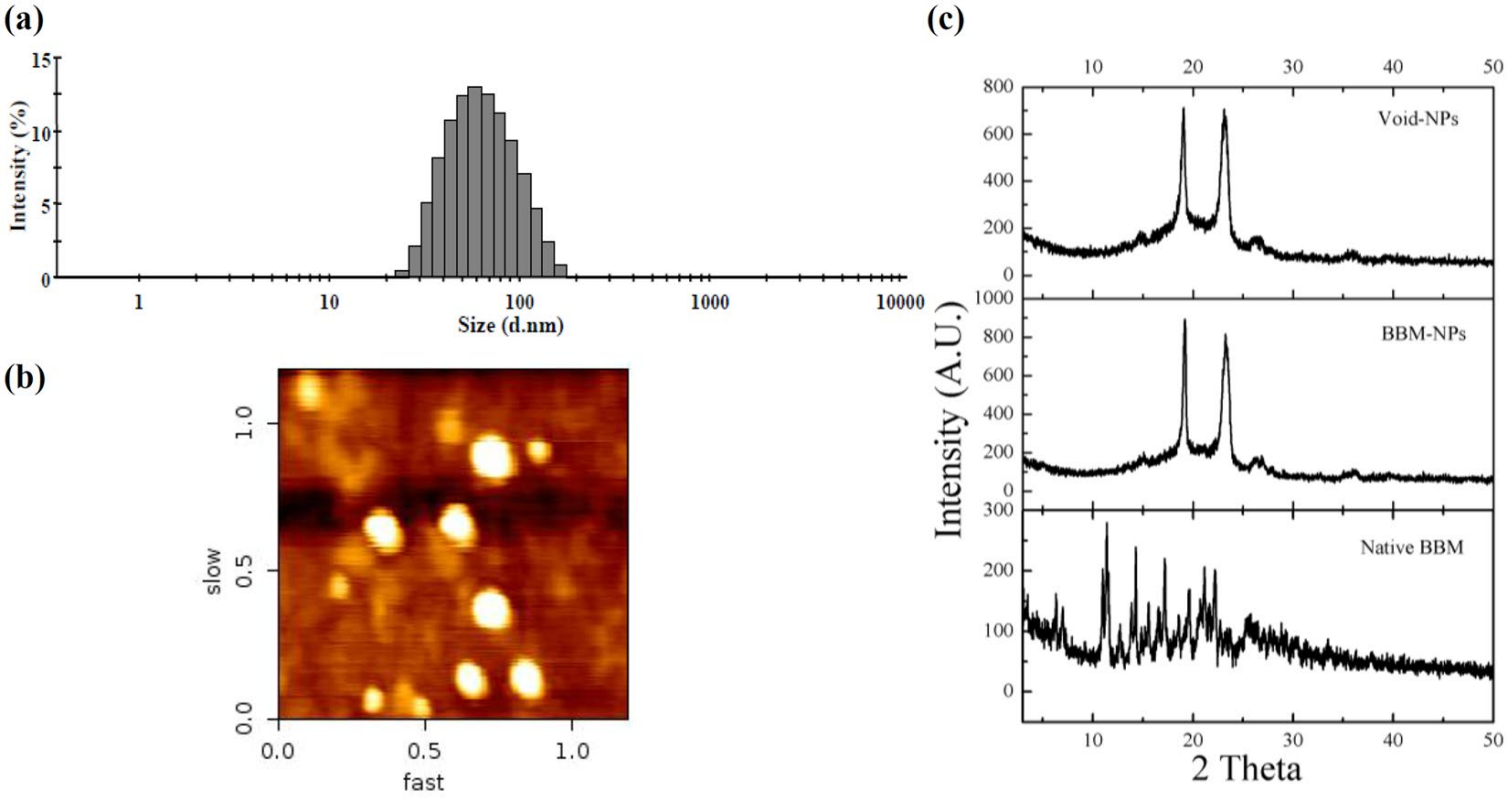
Physico-chemical characterization of BBM-NPs. (**a**) Size distribution of BBM-NPs measured by zetasizer (n = 3). (**b**) The representative picture of BBM-NPs by atomic force microscopy (AFM).(**c**) XRD analysis of Void-NPs, BBM-NPs and Native BBM.

Physico-chemical characterization of BBM-NPs exhibited a unimodal size distribution with a mean hydrodynamic diameter of 75 nm (Fig. 1a) with a negative surface charge (zeta potential = −16 mV) as observed by DLS measurements. The surface topology of BBM-NPs was found to be smooth and spherical as obtained by AFM study (Fig. 1b). HPLC analysis revealed that approximately 250 µg of BBM was encapsulated per mg of NPs (entrapment efficiency 87%). The physical state of the drug inside the NPs was evaluated through XRD analysis. Results revealed that the crystalline peaks obtained in case of native BBM are not present in case of BBM-NPs suggesting that BBM is in amorphous or disordered crystalline phase in the polymer matrix of NPs (Fig. 1c).

### Cellular uptake study

**Figure 2:**
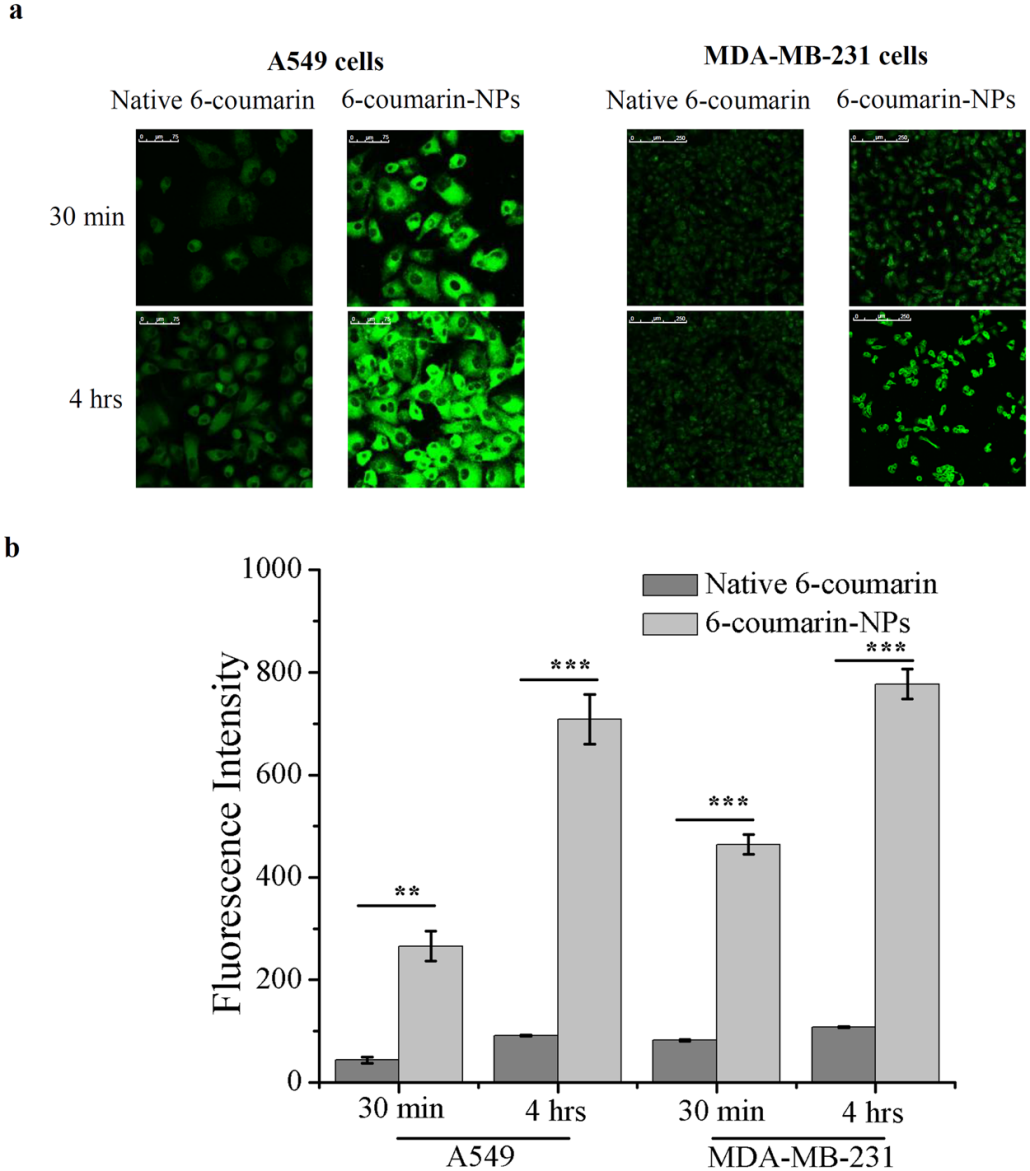
Cellular uptake study of Native 6-coumarin, 6-coumarin-NPs in different cell lines by confocal microscopy and fluorescence spectrophotometer. (**a**) Qualitative cellular uptake study of Native 6-coumarin and 6-coumarin-NPs (50 ng/ml) after 0.5 hr and 4 hrs of incubation in A549 and MDA-MB-231 cells by confocal microscopy. The experiment was done three times and a representative image is provided. (**b**) Quantitative cellular uptake study of Native 6-coumarin and 6-coumarin-NPs (40 ng/ml) after 0.5 hr and 4 hrs of incubation in A549 and MDA-MB-231 cells by fluorescence spectrophotometer (Values are as mean ± SEM, n = 3, **p < 0.001, ***p < 0.0001 6-coumarin-NPs vs. Native 6-coumarin).

The cellular uptake of native 6-coumarin and 6-coumarin-NPs was studied in A549 and MDA-MB-231 cell lines for different time periods. Qualitative cellular uptake study (through confocal microscopy) and quantitative cellular uptake study (through fluorescence spectrophotometer), revealed an augmented cellular uptake of 6-coumarin-NPs as compared to native 6-coumarin in A549 and MDA-MB-231 cell lines in different time periods (i.e. 30 min and 4 hrs) (Fig. 2).

### Cell proliferation assay

**Figure 3:**
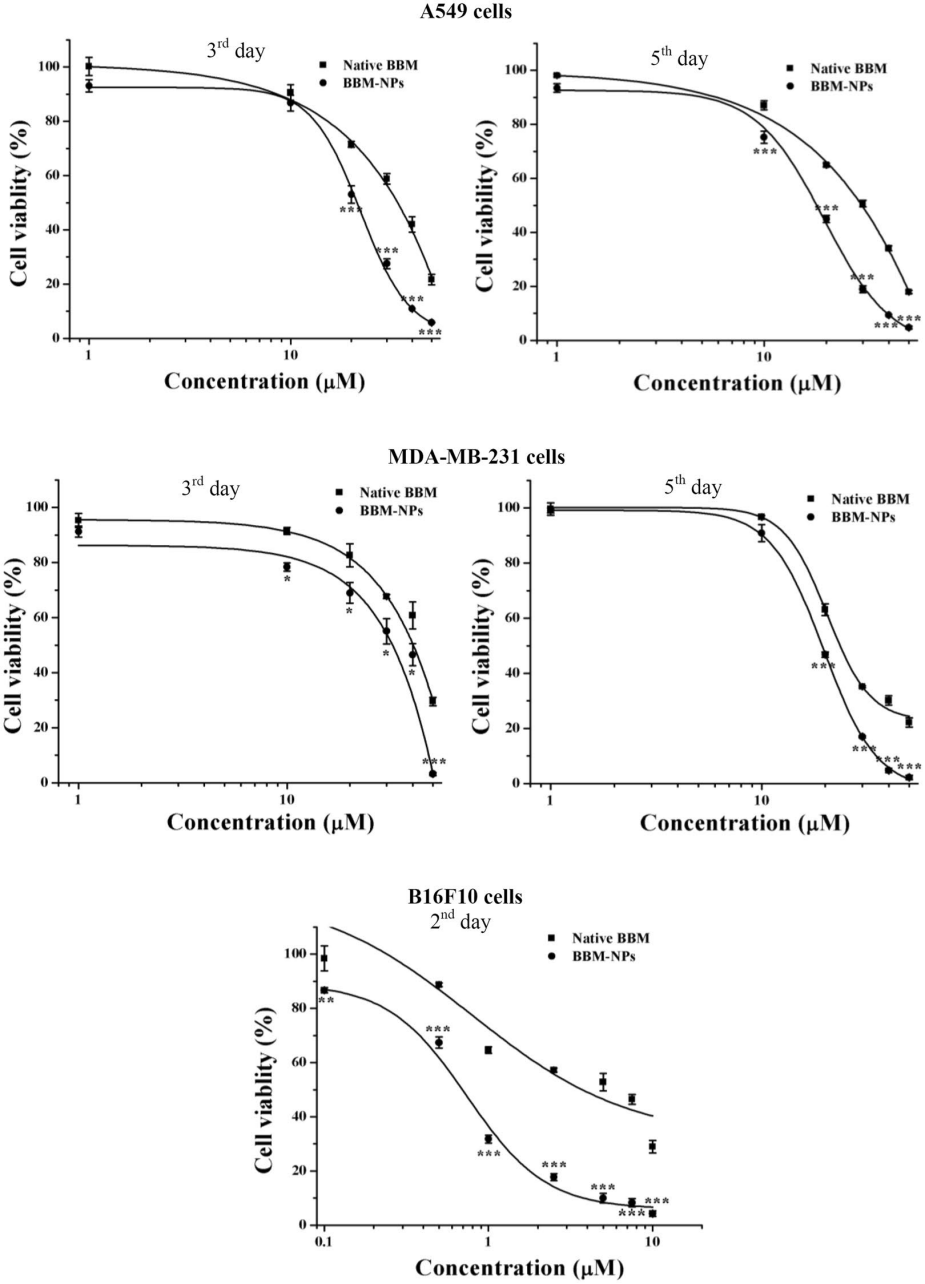
Dose dependent cytotoxicity study of Native BBM, BBM-NPs in A549 and MDA-MB-231 cells after 3^rd^ and 5^th^ day and in B16F10 cells after 2^nd^ day of different treatments by MTT assay. Values are expressed as mean ± SD, n = 4, *p < 0.01, **p < 0.001, ***p < 0.0001 BBM-NPs vs. Native BBM.

Cytotoxicity results illustrated an augmented cytotoxic effect of BBM-NPs as compared to native BBM in A549, MDA-MB-231and B16F10 cells after different days of drug treatment (Fig. 3). The IC_50_ values of native BBM and BBM-NPs calculated from the above study for all the cell lines are shown in Table 1. Results demonstrated that, BBM-NPs was 1.64 & 1.22 times and 1.69 & 1.23 times more effective than native BBM for A549 and MDA-MB-231 cells after 3^rd^ and 5^th^ day respectively and 5.95 times more effective than native BBM for B16F10 cells after 2^nd^ day of treatment. It is also worth mentioning that, the Void-NPs used for this study is non-toxic in nature (data not shown).

**Table 1:** IC_50_ values of native BBM, BBM-NPs in A549. MDA-MB-231 and B16F10 cell lines through MTT assay.

IC_50_ Value (µM)					
Sample	A549		MDA-MB-231		B16F10
	3^rd^ day	5^th^ day	3^rd^ day	5^th^ day	2^nd^ day
Native BBM	34.63 ± 1.12	29.81 ± 0.58	41.94 ± 1.25	23.75 ± 0.44	4.234 ± 0.266
BBM-NPs	21.16 ± 0.89	17.65 ± 0.48	34.14 ± 2.51	19.27 ± 0.25	0.712 ± 0.012

### Inhibition of migration and invasion

**Figure 4:**
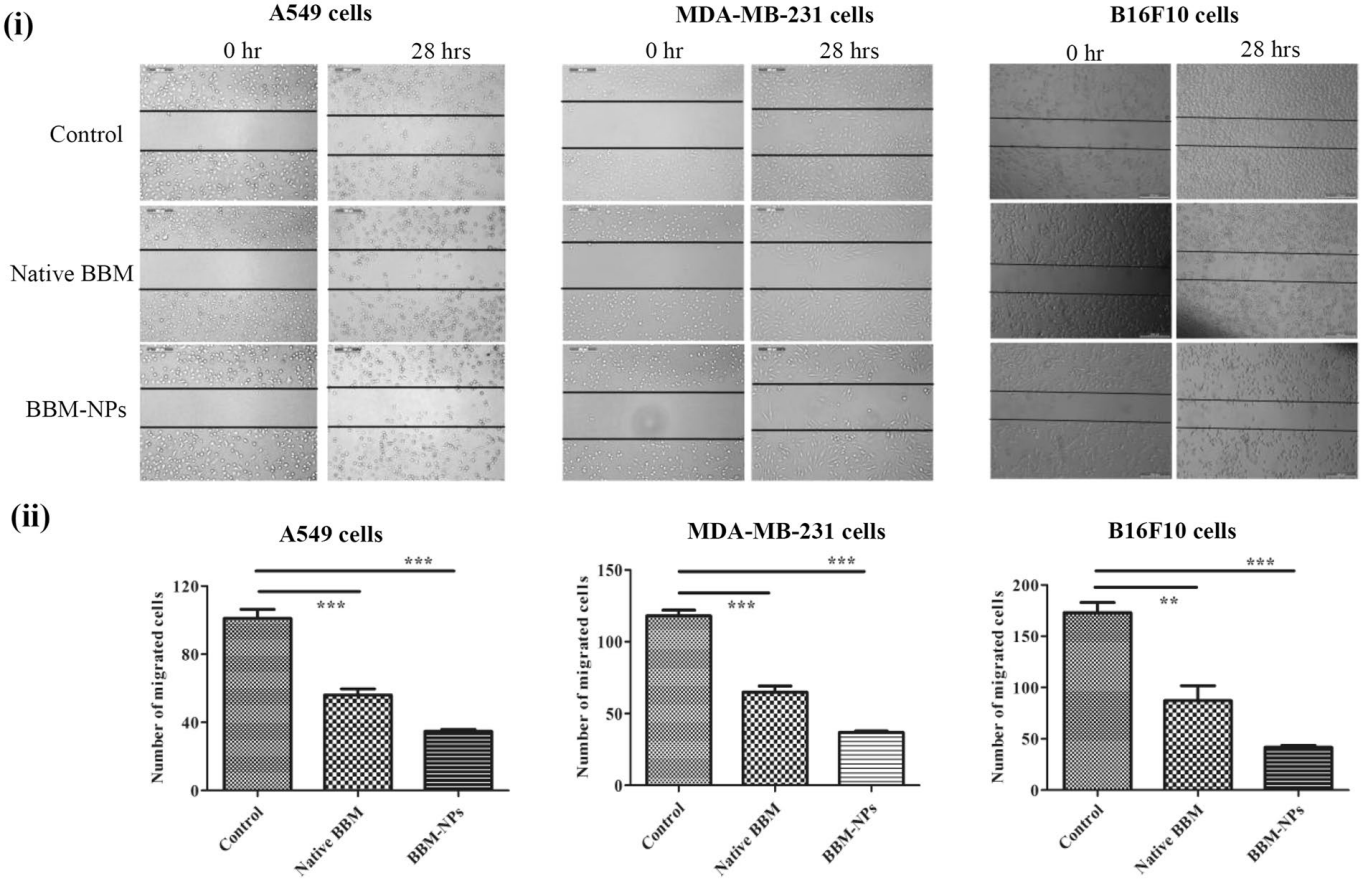
Inhibition analysis of cell migration by a scratch assay in A549, MDA-MB-231 and B16F10 cells by treatment with Native BBM, BBM-NPs (with 1 μg/ml of mitomycin, a proliferation inhibition marker). (**i**) Microscopic picture of migrated cells after 28 hrs. (**ii**) Bar graph represents the number of cells migrated into the scratched area. Values are as mean ± SEM, n = 4, **p < 0.001, ***p < 0.0001 for Native BBM and BBM-NPs vs. control.

**Figure 5:**
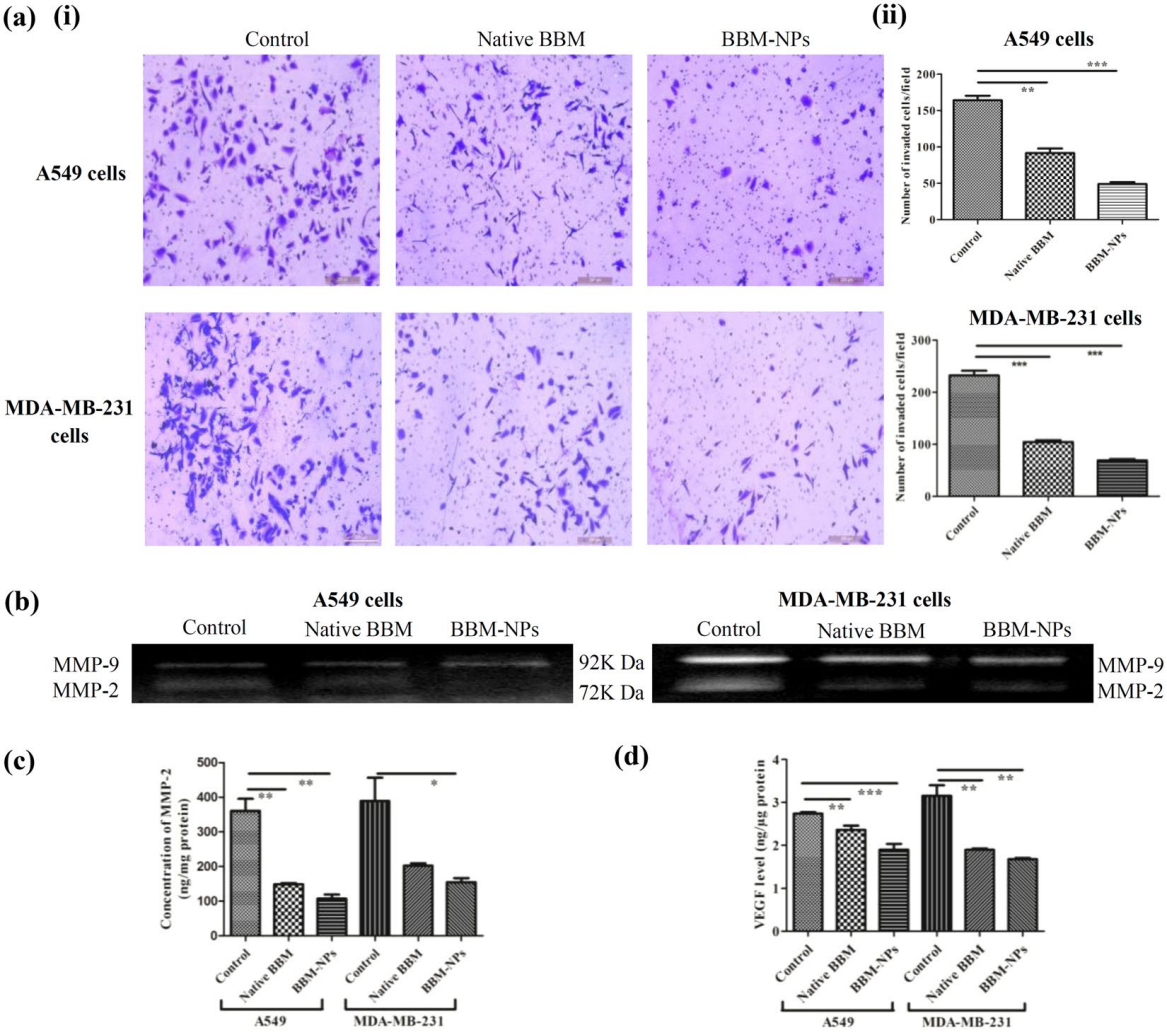
Analysis of cell invasion by using a modified Boyden Chamber assay.(**a**) In brief, A549 and MDA-MB-231 cells at a density 2 × 10^5^ were treated with 25 µM Native BBM/BBM-NPs for 24 hrs. After that, cells were harvested and 1 × 10^5^ live cells seeded in the upper chamber of a matrigel-coated transwell plate and invasion assay was studied by microscopy staining with crystal violet after 24 hrs. (i) Microscopic picture of invaded cells. (ii) Bar graph represents the number of cells invaded the matrigel. Values are as mean ± SEM, n = 3, **p < 0.001, ***p < 0.0001 for Native BBM and BBM-NPs vs. control. During invasion assay, the left over supernatants on the upper chamber of transwell insert was collected from all the treatments of both the cells. (**b**) Inhibition of pro-matrix MMP-9/MMP-2 activation by gelatin zymography (**c**) MMP-2 level measured by ELISA Kit. Values are as mean ± SEM, n = 3, *p < 0.05, **p < 0.005 vs. control. (d) The secreted VEGF in the supernatants measured by ELISA Kit. Values are as mean ± SEM, n = 3, **p < 0.001, ***p < 0.0001 vs. control.

Cancer metastasis mainly occurs due to the migration and invasion of neoplastic cells from a primary tumor to distant sites following angiogenesis and tumor growth. Here, the anti-migratory and anti-invasive effect of BBM was investigated by wound healing assay and Transwell invasion assay. The co-treatment of all the cells with a nontoxic but mitostatic dose of mitomycin rules out involvement of cell proliferation in wound healing assay. In our study, we found that 1 μg/ml concentration of mitomycin inhibited the cell proliferation (data not shown). So, this concentration was used in migration study and results showed that BBM-NPs effectively suppressed the migration of A549, MDA-MB-231 and B16F10 cells as compared to native BBM (Fig. 4). Further, the invasion assay results revealed that at 25 μM, BBM-NPs can efficiently inhibit the invasion of A549 and MDA-MB-231 cells as compared to native BBM (Fig. 5a). During invasion process, matrix metalloproteinases like MMP-2/MMP-9 play an important role as these enzymes degrade the extracellular matrix and basement membrane^18^. Here, gelatin zymography assay results showed that the activation of MMP-2 &MMP-9 was suppressed effectively by BBM-NPs treated cells as compared to native BBM in both the cell lines through the reduction of band intensity (Fig. 5b). The MMP-2 protein level in the supernatants was also measured and results depicted that, in BBM-NPs treated cells, the level of MMP-2 was reduced as compared to native BBM treated cells (Fig. 5c). Further, VEGF has also emerged as an important factor in cancer biology including adhesion, survival, invasion and migration^25^. For this, the VEGF level was measured and results demonstrated that, in both the cell lines, the VEGF expression is lower in BBM-NPs treated case than that of native counterparts (Fig. 5d).

### Mitochondrial function, Apoptosis study and Western blot analysis

**Figure 6:**
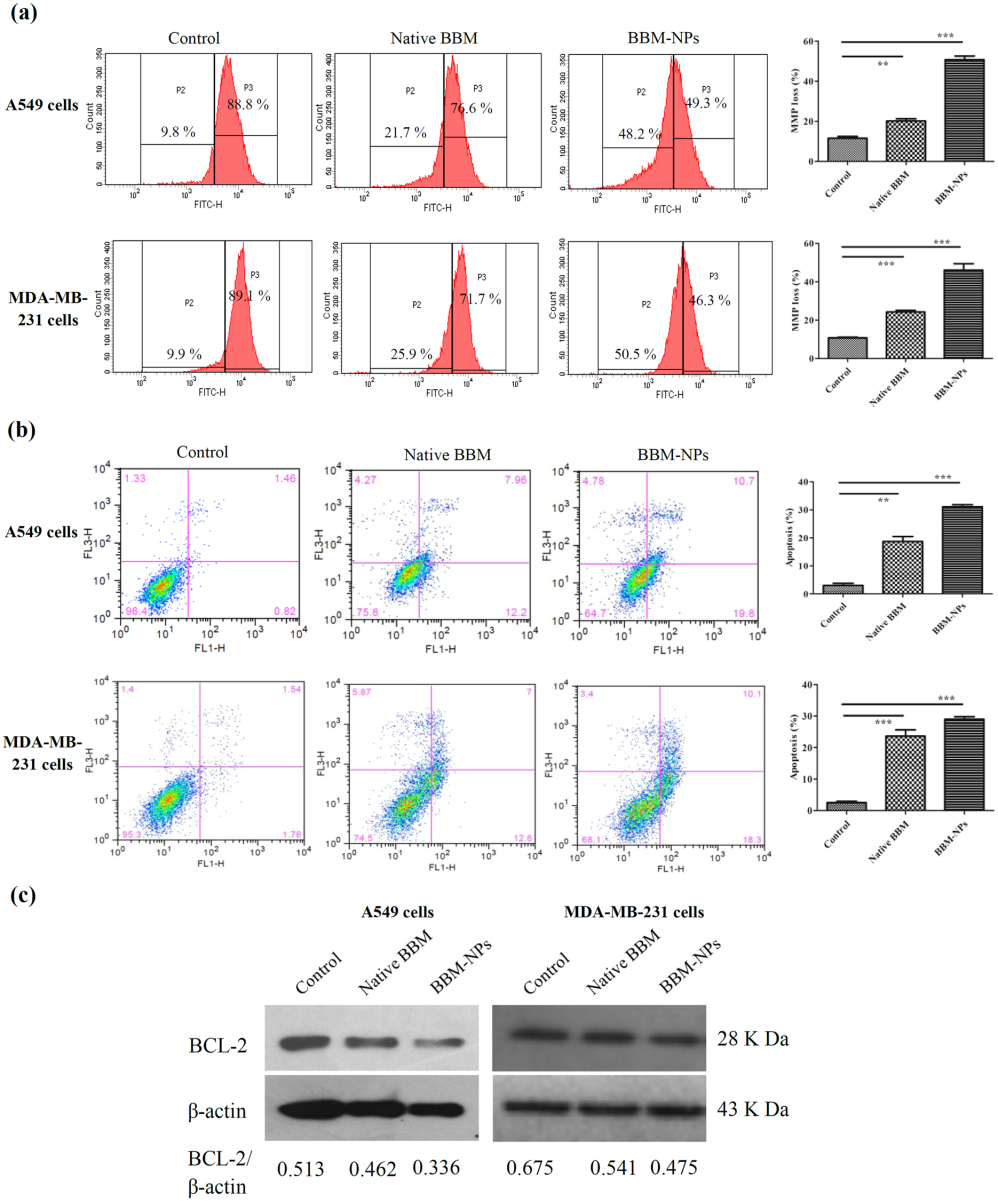
Analysis of mitochondrial function and apoptosis by flow cytometry and expression of anti-apoptotic protein BCL-2 by western blot analysis. (**a**) A549 and MDA-MB-231 cells were treated with Native BBM and BBM-NPs (25 µM) for 48 hrs. Cells associated with mitochondrial function using Rhodamine 123 was analyzed by flow cytometry. Values are as mean ± SEM, n = 3, **p < 0.001, ***p < 0.0001 for Native BBM and BBM-NPs vs. control. (**b**) Induction of apoptosis in A549 and MDA-MB-231 cells treated with Native BBM/BBM-NPs (25 µM) for 48 hrs and percentage of apoptosis (early and late) was measured using Annexin V-FITC by flow cytometry. Values are as mean ± SEM, n = 3, **p < 0.001, ***p < 0.0001 for Native BBM and BBM-NPs vs. control. (**c**) Western blot analysis of anti-apoptotic protein BCL-2 associated with apoptosis in A549 and MDA-MB-231 cell lines following 25 µM BBM treatments (Native/NPs) for 48 hrs. β-actin serves as loading control.

Disruption of active mitochondrion occurs during early apoptosis^26^. The apoptotic cell death induced by BBM is due to the involvement of mitochondrial mediated pathway and for this the mitochondrial function study was performed using Rhodamine 123 through flow cytometry. The mitochondrial function results depicted that treatment with BBM-NPs resulted in rapid dissipation of mitochondrial function due to high level of binding of Rhodamine 123 to mitochondria in cells as observed by decrease of fluorescence intensity as compared to native BBM after 48 hrs of treatment in both A549 and MDA-MB-231 cells (Fig. 6a). Further, BBM is reported to exhibit cytotoxic efficacy by inducing apoptosis following changes in mitochondrial function^16^. The apoptosis results demonstrated that, an augmented apoptotic cell death was observed after BBM-NPs treatment as compared to native BBM in both A549 and MDA-MB-231 cells (Fig. 6b). The mechanistic action of BBM induced apoptosis was further investigated by western blot analysis. Densiometric analysis showed a moderate down regulation of anti-apoptotic protein BCL-2 in BBM-NPs treated cells than that of native BBM treated case in both A549 and MDA-MB-231 cells (Fig. 6c).

### *In vivo* tumor growth study

**Figure 7:**
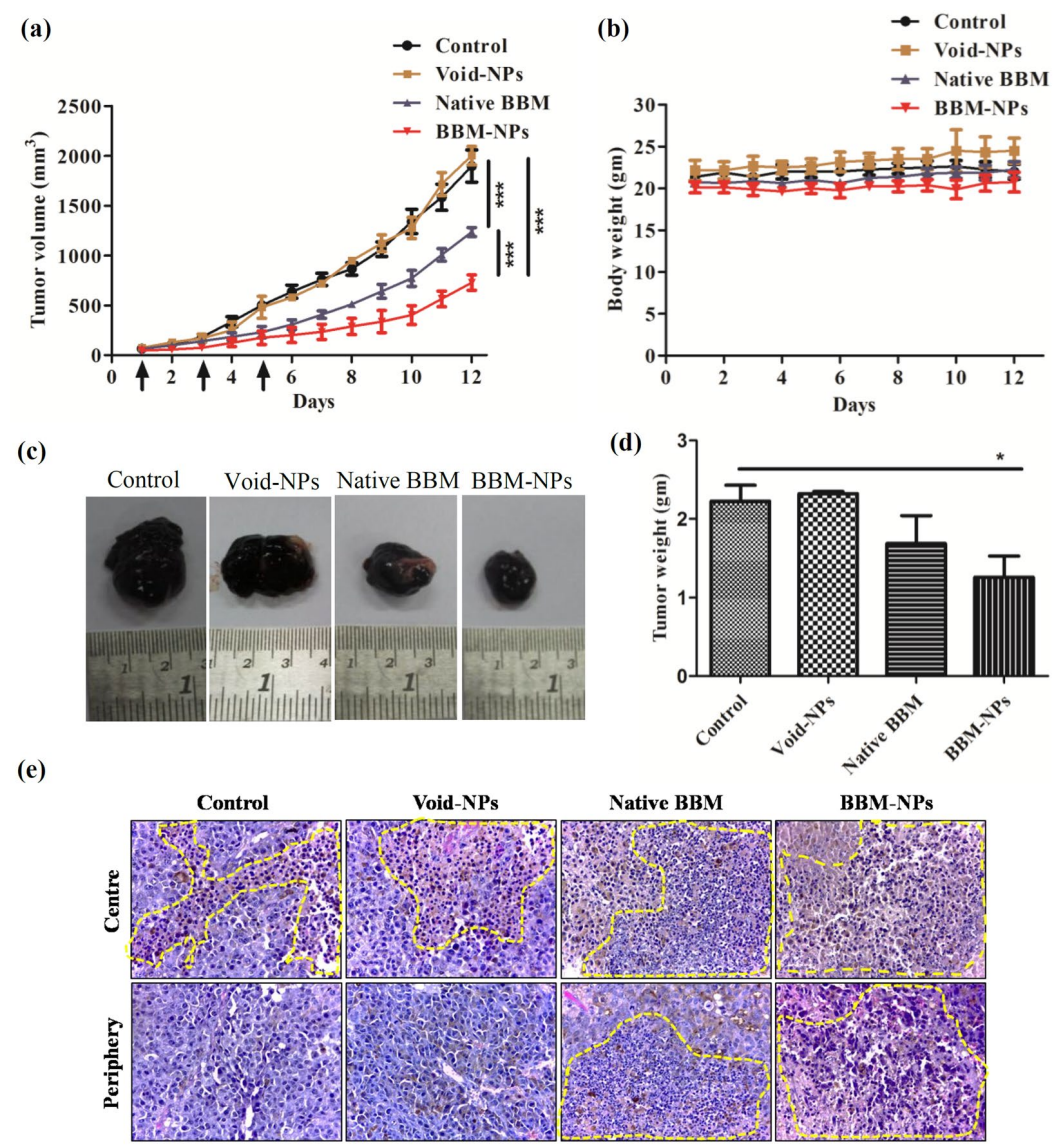
Tumor growth study in C57BL/6 mice melanoma model. The experiment was performed by injecting Native BBM/BBM-NPs/Void-NPs at a dose of 40 mg/kg body weight to mice of different groups intraperitoneally on 1^st^, 3^rd^ and 5^th^ day. (Detailed protocol is in materials and methods). (**a**) Tumor volume measured in mm^3^. (**b**) Body weight change of the mice. (**c**) The representative picture of tumor of different groups. (**d**) Tumor weight measured in grams. (**e**) Representative H&E stained images of B16F10 tumor tissues obtained from different group of animals shows more necrotic area in BBM-NPs treated tumors than in all other groups. Data represented are as mean ± SEM, n = 4, *p < 0.05, ***p < 0.001 for Native BBM and BBM-NPs treated mice vs. control.

This experiment was carried out to investigate the antitumor activity of BBM-NPs in an *in vivo* subcutaneous melanoma (B16F10) tumor model. The tumor volume and body weight were measured daily and finally the tumors were collected after the investigation period from all the groups. There is an exponential growth in the tumor volume of control as well as Void-NPs treated mice. But, the growth of the tumors in case of native BBM and BBM-NPs treated mice was significantly suppressed, BBM-NPs showing more inhibition in tumor growth than native BBM treated mice (Fig. 7a). In all the treatment groups, there were no significant change in the body weight and behavior of the mice during the period of study, which suggests that the treatments do not pose any acute toxicity on the mice (Fig. 7b). The representative picture of tumors as shown in Fig. 7c revealed that, the control and Void-NPs treated mice have almost similar tumor size, whereas BBM-NPs treated mice showed a reduction in tumor size. Further, the weight of the tumors also showed similar results showing significant decrease in tumor weight in case of BBM-NPs treated mice as compared to mice treated with native BBM (Fig. 7d). Furthermore, H&E staining results showed more necrotic area in BBM-NPs treated B16F10 tumors as compared to other groups (Fig. 7e).

### *In vivo* metastatic growth study

**Figure 8:**
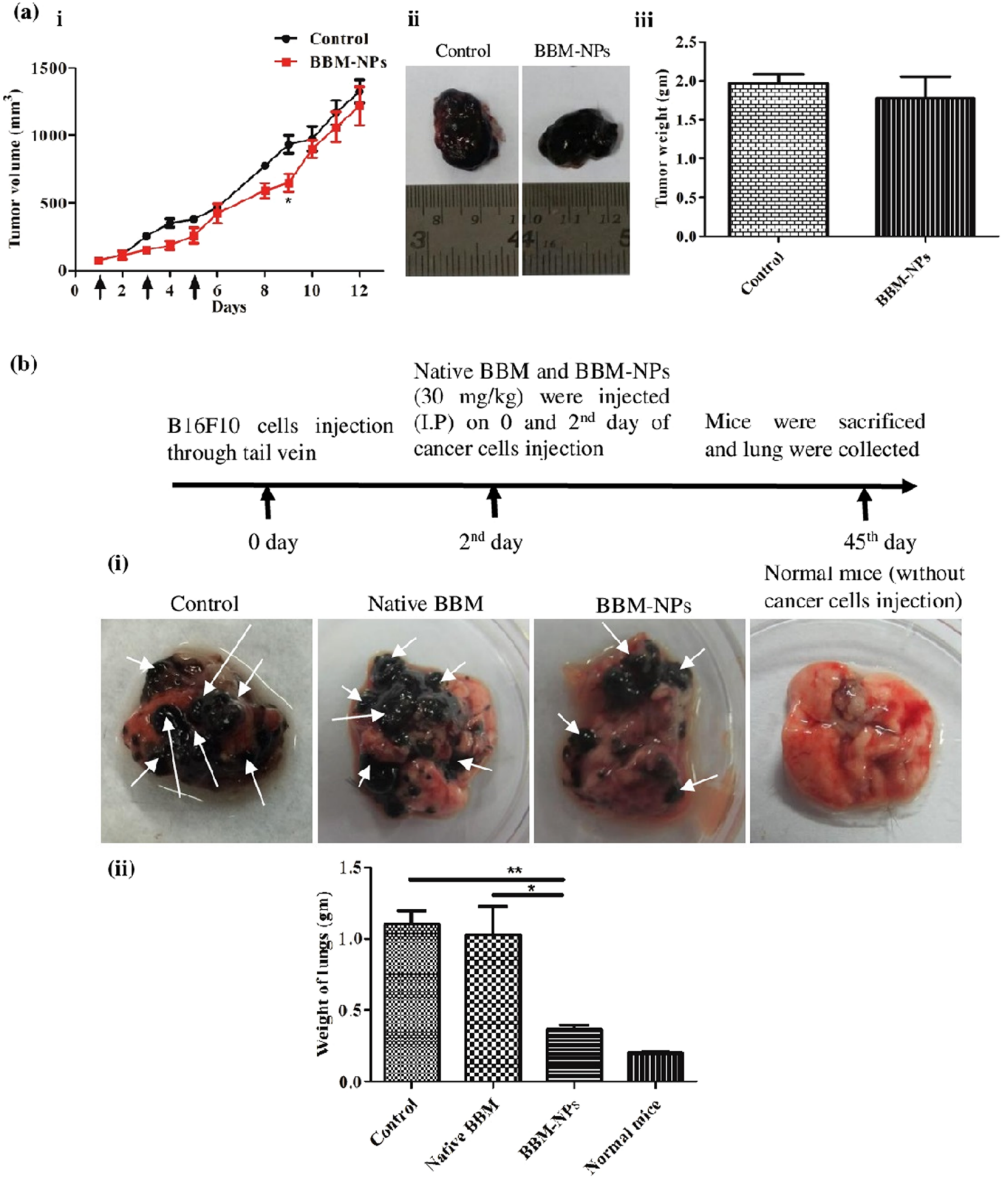
Anti-metastatic study of BBM-NPs in C57BL/6 mice melanoma model. (**a**)To achieve a moderately cytotoxic dose of BBM-NPs to the tumor, initially we have developed a subcutaneous tumor model (detailed protocol in materials and methods) and injected BBM-NPs at a dose of 30 mg/kg body weight on 1^st^, 3^rd^ and 5^th^ day intraperitoneally and finally sacrificed on 12^th^ day. (i) Tumor volume in mm^3^. (ii) The representative picture of the tumor. (iii) Tumor weight measured in grams. Data represented above are as mean ± SEM, n = 3. (**b**) To evaluate the efficacy of BBM to hematogenous lung metastasis, we have injected B16F10 cells (2 × 10^5^) to the tail vein of C57BL/6 mice (n = 3). Next, we have injected Native BBM and BBM-NPs (30 mg/kg body weight) on 0 and 2^nd^ day of cells injection. After 45 days, the mice were sacrificed and lungs were collected and weighed. (i) The representative picture of the lungs. (ii) The weight of lungs in grams. The datas are represented as mean ± SEM, *p < 0.05, **p < 0.005 for BBM-NPs treated mice vs. Native BBM and vs. control. Arrow indicates the tumor nodule.

Based on the *in vitro* observations that BBM-NPs can inhibit cancer cell migration without altering cell death with a dose of native BBM/BBM-NPs (25 µm BBM) (Fig. 5), we were interested to evaluate its antimetastatic effect *in vivo*. To rule out the effect of BBM-mediated cell death on overall metastasis, initially we have checked a lower dose of BBM that has less or moderate cytotoxic effect *in vivo*. In this regard, the study showed that at a dose of 30 mg/kg body weight, BBM-NPs has very less effect on primary tumor growth (Fig. 8a). Further, the metastatic tumor growth study as observed after 45 days of cells injection (I.V) through the tail vein of C57BL/6 mice showed that the BBM-NPs can effectively inhibit lung metastasis as compared to native BBM. The extent of metastasis was evident from the formation of nodules in mouse lung (Fig. 8b). The weight of the lungs was also found to be lower in BBM-NPs treatment than that observed in native BBM treated and control mice. Most importantly, it is noted that our formulated NPs at lower dose than that used in case of tumor growth study can also effectively inhibit the metastasis.

## Discussion

Recently, targeting and inhibiting both tumorigenesis and metastasis have become the main focus in cancer research to achieve effective therapeutic responses. Different chemotherapeutic agents are used for the treatment of cancer. However, these agents often display limited activity on metastasis. The current therapeutics which mostly targets cancer cells in their active growth and division cycle are ineffective against the slowly growing micrometastasis. Further, presence of intrinsically more drug-resistant cancer cells in metastatic tumors, also limits the success of chemotherapy^7, 27–29^. Recently, BBM has shown its potent antitumor activity in different types of cancer including metastatic cancer with reduced toxicity^17, 18, 30, 31^. Though BBM is a potent drug but its half-life in blood plasma is very short, owing to its quick renal clearance^32^. In this context, nanotechnology based drug delivery vehicles are regarded as an emerging approach in cancer therapy as they are able to overcome the above problem^19, 33, 34^. Further, the bilayer structure of lipid based NPs with excellent biocompatibility, biodegradability and nontoxicity nature, have led them to be used as a favorable platform for achieving successful delivery of anticancer drugs in a sustained manner^28^.

The formulated BBM-NPs of size ~100 nm with negative surface charge (Fig. 1),could evade the reticuloendothelial system by escaping macrophage uptake, resulting in enhanced accumulation of drug at the target site over a period of time, which ultimately facilitates prolonged cytotoxicity^35–37^. During the preparation of lipid based NPs, we have used pluronic F127 as a stabilizer which not only preserves the integrity of the internal structure of the particles but also enhances colloidal stability^38^. Enhanced internalization of drug loaded NPs by the cells is also a prerequisite for an efficient cancer therapeutic approach^39^. In our study, both qualitative and quantitative uptake analysis revealed the higher uptake of 6-coumarin-NPs in both A549 and MDA-MB-231 cells (Fig. 2), which may be due to the entry of NPs through endocytosis whereas the native drug gets internalized by passive diffusion^23, 40^. Moreover, the higher uptake of NPs may be responsible for obtaining superior cytotoxicity (Fig. 3, Table 1). Metastasis process involves invasion and migration of cancer cells and enzymes like MMP-2/MMP-9 play a major role in this process. In this regard, Wang *et al*. have reported that BBM can effectively suppress the growth, invasion and migration of highly metastasized breast cancer cells by reducing the promatrix MMP-2/MMP-9 in the supernatants of invading MDA-MB-231 cells^18^. In our study, we have also found that the BBM-NPs can effectively inhibit migration and invasion which might be due to reduced MMP-2/MMP-9 activation and VEGF expression in highly metastatic cancer cells in comparison to native BBM (Figs. 4 and 5). Further, a study conducted by Wang *et al*. showed that BBM has antiproliferative effects with cell cycle arrest ability leading to mitochondrial transmembrane potential loss (MTP loss), caspase activation and apoptotic cell death in human hepatoma cell line SMMC7721^16^. In our study, BBM-NPs showed higher percentage of cells associated with mitochondrial function and apoptotic cell death than that of native BBM treatments (Fig. 6a–c). The superior cell death and apoptosis induction ability as observed by BBM-NPs may be due to the enhanced accumulation of drug at the tumor site with sustained release phenomenon^41, 42^.

The tumor growth study results demonstrated that the BBM-NPs can effectively reduce the tumor volume and weight in comparison to native BBM treated mice. The Void-NPs treated mice and control mice have shown similar type of tumor weight and tumor volume (Fig. 7a,c,d). Further, absence of significant loss of body weight and behaviour (physical activity) of mice suggests that BBM (both native/NPs) treatments do not have any acute toxicity to the mice (Fig. 7b). In a study conducted by Wang *et al*. it is reported that in nude mice, BBM can effectively inhibit the tumor growth without having any toxic side effects at a dose of 40 and 80 mg/kg body weight intraperitoneally^43^. The higher effectiveness of BBM-NPs may be attributed to the enhanced accretion of nanoparticulate drug at the tumor site with sustained release over a period of time, due to EPR effect^44, 45^. In another study, Wu *et al*. have shown that micellar nanocarriers incorporating (1,2-diamino cyclohexane) platinum (II) (DACHPt) effectively inhibited the primary tumor growth and overt liver metastases by EPR effect and also can target early preangiogenic metastases based on inflammatory microenvironment of the metastatic niche^46^. Further, Athawale *et al*. have reported that Etoposide loaded solid lipid nanoparticles (Et-SLN) treatment demonstrated a significant reduction in the lung metastasized tumor colonies due to high accumulation of etoposide in the lung and liver with SLN^47^.

Our *in vitro* migration study clearly suggested the effect of BBM on inhibiting metastatic events of cancer cells at molecular and cellular level (Fig. 5). However, in many studies the antimetastatic effect of cytotoxic drugs are due to nonspecific cytotoxicity of the drugs rather than actual effect on cancer cell metastasis process^48^. Here, we have evaluated the effect of a noncytotoxic dose of BBM on hematogenous metastasis of B16F10 cells. B16F10 mouse melanoma cells were taken for developing *in vivo* metastatic tumor model due to their high metastatic potential. Moreover, it is regarded as a useful model to study hematogeneous lung metastasis^49^. Further presence of black pigments (melanin) gives added advantage to detect even small tumor nodules with naked eye. Though hematogenous lung metastasis after I.V. injection of melanoma cells through tail vein, does not recapitulate all the events of actual metastasis, but it can mimic certain important steps of metastasis like extravasation of cancer cells into lung parenchyma, their survival in the lung tissue and local invasion. Extravasation of cancer cells might occur within 24 hrs of its injection into blood circulation. Therefore, we thought to inject drug at the same time after I.V. injection of melanoma cells. The efficacy of BBM-NPs to reduce the metastatic tumor burden in lungs without significantly altering the growth of primary tumor suggests that BBM-NPs could inhibit some critical cellular and molecular events in metastasis. Based on our *in vitro* data, this inhibition of metastasis through BBM-NPs could be due to inhibition of MMPs and growth factors like VEGF (Figs 5c,d and 8). Recently, Kopp *et al*. have shown the effectiveness of Salinomycin treatment on cancer cell migration with reduced metastatic tumor burden, at a dose with minor cytotoxicity in a syngeneic mouse model^48^. In this regard, the primary tumor growth study revealed that BBM-NPs at a dose of 30 mg/kg body weight have very less cytotoxic effect on tumor cells and it is found that this dose can effectively inhibit lung metastasis. Importantly, similar tumor size in both control and treated animals but low tumor burden only in NPs treated animals further signifies the importance of non cytotoxic dose of BBM in inhibiting the metastasis events. In this context, it will be appropriate to mention that some commonly used chemotherapeutic drugs like gemcitabine and paclitaxel has shown to promote metastasis at a nontoxic or lower dose^50, 51^. Therefore, the current data suggests the clinical significance of low dose BBM as an antimetastatic therapeutic.

## Conclusion

Development of different strategies using nano-drug delivery platforms for selective delivery of drugs at the tumor site through EPR effect with reduced toxicity is urgently required for the treatment of primary tumor growth and metastasis in most of the cancer patients. In this milieu, the current work focused on to formulate efficient BBM-NPs for the treatment of metastatic cancer in both *in vitro* and *in vivo*. The formulated NPs showed better intracellular uptake than that of native counterpart. Further, the results showed that BBM-NPs can effectively inhibit cell proliferation, invasion, and migration along with higher percentage of cells associated with mitochondrial function followed by apoptosis and down regulation of anti-apoptotic protein BCL-2. Furthermore, tumor growth study indicated that BBM-NPs can effectively reduce the tumor growth and tumor volume in comparison to native BBM treated mice. Finally, BBM-NPs effectively inhibited the formation of lung metastasized nodules than that of native BBM treatment. Thus, BBM-NPs might be a potential therapeutic approach against multiple malignancies.

## Methods

### Materials

Berbamine (BBM), Mitomycin C, Pluronic F-127, poly (ethylene glycol)-10,000 (PEG – 10,000), Rhodamine 123, Tween-80, propidium iodide (PI), 6-coumarin, protease inhibitor cocktail, sodium dodecyl sulphate (SDS), glycine, 3-(4, 5-dimethylthiazol-2-yl)-2, 5-diphenyl tetrazolium bromide (MTT), D-α-Tocopherol poly (ethylene glycol) 1000 succinate (TPGS), bradford reagent, triethyl amine (TEA), crystal violet, gelatin, brilliant blue G, p-coumaric acid and luminol were procured from Sigma-Aldrich (St. Louis, MO). Glycerylmonooleate (GMO) was purchased from Eastman (Memphis, TN). Sodium chloride was procured from MP biomedical (Cedex, France). Acetonitrile and methanol were obtained from Spectrochem, Pvt. Ltd. (Mumbai, India). Dimethyl sulphoxide, ammonium acetate and acetic acid were obtained from Merck, India Pvt. Ltd. (Mumbai, India). Tris base was purchased from Promega (Promega Corporation, Madison, Wisconsin). All other chemicals used were purchased from Sigma-Aldrich (St. Louis, MO) and used without further purification.

### Cell culture

A549 lung cancer, MDA-MB-231 breast cancer and B16F10 mouse melanoma cell lines were obtained from National Centre for Cell Sciences (NCCS), Pune, India and were cultured in DMEM (PAN BIOTECH GmbH, Aidenbach,Germany) with 10% fetal bovine serum (FBS), supplemented with 1% L-glutamine and 1% penicillin - streptomycin (Himedia Laboratories Pvt. Ltd., Mumbai, India). The maintenance of cells was done at 37 °C in a humidified, 5% CO_2_ atmosphere in an incubator (Hera Cell, Thermo Scientific,Waltham, MA).

### Preparation of NPs

Preparation of BBM-NPs and 6-coumarin loaded NPs (6-coumarin-NPs) were done by following our previous protocol with slight modification^22^ (Supplementary section).

### Physico-chemical characterization of NPs

Based on dynamic light scattering, the particle size and size distribution measurements of BBM-NPs were done by using Malvern Zetasizer Nano ZS (Malvern Instrument, UK) following our previously published protocol. All measurements were performed in triplicates. The analysis of size, shape and surface properties of BBM-NPs were analyzed by Atomic Force Microscopy (AFM) (JPK nanowizard II, JPK instrument, Bouchestrasse, Berlin, Germany) using our previously published protocol^22^.

### X-ray powder diffraction (XRD) Study

XRD analysis was carried out to know the crystalline peak of the drug and its physical state inside the NPs. Native BBM, BBM-NPs and Void-NPs were taken for XRD analysis by using X-ray diffractometer (D8 ADVANCE, Bruker, Madison, WI). The measurements were done at a voltage 40 KV and 40 mA. The scanned angle was set from 3° ≤ 2θ ≤ 50° at the scan rate 3°/min.

### Determination of entrapment efficiency of BBM

The amount of BBM encapsulated into the NPs was quantified by using reverse phase isocratic mode of high performance liquid chromatography (RP-HPLC) system, Waters ^TM^ 600 (Waters Co., Milford, MA) as reported by Fan *et al*.^52^ (Supplementary section).

### Cellular uptake studies

Qualitative and quantitative cellular uptake study of native 6-coumarin, 6-coumarin-NPs were carried out in A549 and MDA-MB-231 cell lines by confocal microscopy (concentration 50 ng/ml) and fluorescence spectrophotometer (concentration 40 ng/ml) respectively following our previously published protocol^22^ (Supplementary section).

### Cell proliferation assay

The cytotoxic effect of native BBM and BBM-NPs was investigated by MTT assay in A549, MDA-MB-231 and B16F10 cell lines using our previously published protocol^53^ (Supplementary section).

### Migration assay

Migration and invasion plays an important role during cancer metastasis, where neoplastic progression occurs from primary tumor to distant sites. So, a scratch assay, known as wound healing assay was carried out to check the migration of cells to the scratch area by following the protocol of Senft *et al*.^54^ with little modifications. Briefly, 2 × 10^5^ cells/well in a 6 well plate with 80% sub-confluence, were treated with 25 μM concentration of native/nano formulation for A549 & MDA-MB-231 cells and 2.5 μM concentration for B16F10 cells for 2 hrs and then migration assay was performed (Supplementary section).

### Invasion assay

The invasion properties of tumor cells were analyzed using the Transwell cell culture chamber (BD BIOCOAT Cell culture Insert, Bedford, MA) with 8 µm pores as described by Senft *et al*. with slight modifications^54^. In brief, 2 × 10^5^ A549 and MDA-MB-231 cells/well were treated with 25 μM concentration of BBM or equivalent concentration of BBM-NPs for 24 hrs. After trypsinization, 1 × 10^5^ live cells in serum free media were seeded into the matrigel coated transwell inserts. In the lower chamber of the inserts, DMEM media with serum was added and incubated for 24 hrs at 37 °C. Then, the supernatants were collected from the upper chamber to check the matrix metalloproteinase-2/9 (MMP-2/MMP-9) and vascular endothelial growth factor (VEGF) secreted by the cells to invade through the matrigel and invasion assay was performed. MMP-2/MMP-9 activation was assessed by gelatin zymography and the levels of MMP-2 and VEGF in the above supernatants were checked through ELISA Kit (Supplementary section).

### Mitochondrial function study

The changes in the mitochondrial function was evaluated by flow cytometer using rhodamine 123 dye^43^. In brief, 2 × 10^5^ A549 and MDA-MB-231 cells were treated with 25 μM BBM or equivalent concentration of BBM-NPs for 48 hrs (Supplementary section).

### Apoptosis study by Flow Cytometry

The percentage of apoptosis in A549 and MDA-MB-231 cells after treatment with native BBM and BBM-NPs were analyzed by flow cytometry^43^. In brief, 2 × 10^5^ cells/well were treated with 25 μM native/nanoformulation for 48 hrs (Supplementary section).

### Western blot analysis

Western blot analysis was undertaken to know the molecular mechanism of apoptosis modulated by BBM^18^. In brief, A549 and MDA-MB-231 cells (2.5 × 10^5^) were treated with 25 μM concentration of BBM native/nanoformulation for 48 hrs. After that, cells were collected by scraping, washed with cold PBS followed by protein isolation and estimation following our previously published protocol^55^. Western blot analysis of proteins were performed using specific primary antibody for recognizing β-actin (Santa Cruz Biotechnology, Inc., Santa Cruz, CA) and BCL-2 (Cell signalling Technology, Inc., Danver, MA.) using our previously published protocol^55^.

### *In vivo* tumor growth study

*In vivo* experiments were performed with the permission of the Institutional Animal Ethics Committee (IAEC) of the Institute of Life Sciences, Bhubaneswar, India. All experiments were performed in accordance with relevant guidelines and regulations. Female C57BL/6 mice of 6-8 week’s old were taken for the experiments. Briefly, 3 × 10^5^ B16F10 mouse melanoma cells were injected subcutaneously in 100 μl DPBS on the left flank of the mouse. After formation of tumor with volume around 50–70 mm^3^, mice were randomly divided into four groups (n = 4). The first group was taken as control and second, third & fourth groups were administered with Void-NPs, native BBM & BBM-NPs respectively at a dose 40 mg/kg body weight intraperitoneally (I.P.) on 1^st^, 3^rd^ & 5^th^ day. The tumor volume and body weight of the mice were measured daily during the period of investigation. Values of tumor volume (v) were determined by measuring the longitudinal cross section (l) and transverse section (w) by a digital slide calipers and then applying the formula $v=1/2\,{w}^{2}\times 1$. Finally, the mice were sacrificed, tumors were harvested, photographed and weighed in a digital balance. Further, H&E staining was performed by taking the tumor tissues obtained from different group of animals to evaluate necrosis.

### *In vivo* metastasis study

For metastatic study, a lower dose of drug is required, which will be moderately cytotoxic to the cancer cells but can effectively inhibit metastasis^48^. So, before performing the metastasis study, first a lower dose of BBM than the dose used during tumor growth study was selected and its cytotoxicity was checked in subcutaneous melanoma tumor model. In brief, the animal having subcutaneous tumor volume of 50-70 mm^3^ were divided into two groups (n = 3), one group served as control and another group was treated with BBM-NPs at a dose of 30 mg/kg body weight intraperitoneally on 1^st^, 3^rd^ and 5^th^ day. The tumor volume and body weight was measured daily during the period of study. Then the animals were sacrificed and tumors were collected, weighed using a digital balance.

Thereafter, the metastasis study was carried out. For this, 2 × 10^5^ B16F10 cells suspended in 200 µl of DPBS was injected intravenously and randomly assigned to three different groups (n = 3). The injected B16F10 melanoma cells spontaneously metastasized to the mouse lung and then I.P. injection of native BBM/BBM-NPs were given on 0 and 2^nd^ day of cells injection at a dose of 30 mg/kg body weight. After 45 days, the mice were sacrificed and immediately the mouse lung was collected and weighed using a digital balance. The plot was made to compare the weight of lungs with or without drug treatment.

### Statistical analysis

Two way analysis of variance (two way ANOVA) and student’s *t* test were performed to conduct statistical analysis. Data are expressed as mean ± standard error of mean (SEM) or standard deviation (SD) and values of *p* < 0.05 were indicative of significant differences.

## Electronic supplementary material

Supplimentary Info
