# Supplementary material for: Enhanced anti-metastatic and anti-tumorigenic efficacy of Berbamine loaded lipid nanoparticles in vivo
Source: Sci Rep. 2017 Jul 19;7:5806. doi: 10.1038/s41598-017-05296-y (PMC5517447; doi:10.1038/s41598-017-05296-y)
Supplement: Supplementary file 1 — Supplimentary Info [file 41598_2017_5296_MOESM1_ESM.doc]

**Enhanced anti-metastatic and anti-tumorigenic efficacy of Berbamine loaded lipid nanoparticles *in vivo***

Priyambada Parhi^1^, Sujit Suklabaidya^1, 2^ and Sanjeeb Kumar Sahoo*^1^

^1^Institute of Life Sciences, Nalco Square, Chandrasekharpur, Bhubaneswar, India.

^2^ Manipal University, Karnataka, India.

**Corresponding Author ***

Sanjeeb K. Sahoo, Ph.D

Nanomedicine Laboratory

Institute of Life Sciences

Nalco Square, Chandrasekharpur

Bhubaneswar, Orissa, 751 023, INDIA

E-mail- sanjeebsahoo2005@gmail.com

Phone- +91-674-2302094

Fax- +91-674-230072

**Supplementary Methods**

**Preparation of NPs**

Briefly, 60 mg BBM was incorporated into the fluid phase of GMO (50 µl at 40 °C) and vortexed. After that emulsification of the above mixture was done by adding 1 ml of pluronic F-127 solution (5 % w/v) followed by sonication, using a microtip probe sonicator (Model: VC 505 Vibracell Sonics, Newton) for 2 min at amplitude 30 % over an ice bath and the above solution was further emulsified with 1 ml of TPGS (5 % w/v) and sonicated. The rest procedure including lyophilization was done by following our previously published protocol ^1^. To determine the cellular uptake of NPs, in a similar way, 6-coumarin loaded NPs (6-coumarin-NPs) were formulated by following above protocol except the addition of 100 µg of the dye to GMO prior to emulsion instead of BBM.

**Determination of entrapment efficiency of BBM**

Briefly, BBM-NPs were dissolved in acetonitrile (~ 1 mg/ml) and then sonicated for 2 min at 30 % amplitude in an ice bath (Model: VC 505 Vibracell Sonics, Newton) and centrifuged at 13,800 rpm for 10 min at 4 °C (SIGMA 1-15K, Germany). The drug content was measured through RP-HPLC by injecting 50 µl of the above supernatant using a mobile phase of acetonitrile (32 %): Buffer (68 %) (v/v). The buffer made up of 30 mM ammonium acetate and 14 mM TEA, pH adjusted to 4.85 with acetic acid. The flow rate was set at 1 ml/min with a quaternary pump (M600E WATERS ^TM)^ at 30 °C having C18 column (Nova – Pac, 3.9 mm X 300 mm, Waters associates, Milford, MA). Quantification of BBM level was done by UV detection at 254 nm with dual wavelength detector (M2489). The amount of BBM in NPs was determined from the peak area correlated with the standard curve. The standard curve of BBM was prepared under identical condition.

**Cellular uptake studies**

6-coumarin was taken as a fluorescent probe to study the intracellular uptake and retention due to its high fluorescent activity and offer a sensitive method to quantitatively determine the intracellular uptake ^2^. Qualitative and quantitative cellular uptake study of native 6-coumarin, 6-coumarin-NPs were carried out in A549 and MDA-MB-231 cell lines by confocal microscopy (concentration used 50 ng/ml) and fluorescence spectrophotometer (concentration used 40 ng/ml) respectively following our previously published protocol ^1^. Both qualitative and quantitative cellular uptake was performed after 30 min and 4 hrs incubation. Qualitative uptake study done by confocal microscopy was repeated thrice and representative picture of a single experiment has been provided. Quantitative uptake study was done in triplicates and data represented as mean ± SEM.

**Cell proliferation assay**

Briefly, 2000 cells/well of A549, MDA-MB-231 and B16F10 cell lines were seeded in 96 well plates (Corning, NY). Next day, the cells were treated with different concentrations of either native BBM or equivalent concentration of BBM-NPs. Equivalent amount of Void NPs (without drug) was used to evaluate the toxicity of Void-NPs. Medium treated cells were taken as control for the experiment. MTT assay was carried out on 3^rd^ and 5^th^ day in case of A549 and MDA-MB-231 cells and on 2^nd^ day in case of B16F10 cells ^3^. The cytotoxic effects were measured as a percentage of cell growth with respect to respective control. The data represented as mean ± SEM, (n = 4). The IC_50_ (Inhibitory concentration to kill 50 % of cells) value was calculated by nonlinear regression analysis using the sigmoid plot equation ^4^.

**Migration assay**

Briefly, the cells seeded at a density 2 × 10^5^ cells/well in a 6 well plate after reaching 80 % subconfluency, were treated with 25 μM concentration of native BBM or equivalent concentration of BBM-NPs for A549 & MDA-MB-231 cells and 2.5 μM concentration for B16F10 cells for 2 hrs. Untreated cells were considered as control. After that, the media was replaced with fresh media with 1 μg/ml mitomycin (a known inhibitor of proliferation) and a scratch was made by using sterile 2.5 μl tips and marked. The cells were washed with PBS and pictures of migrated cells in the scratched area were taken in 0 hr and 28 hrs after making the scratch, by using inverted phase contrast microscope (Leica QWin, UK) with 10X objective and number of cells migrated to the scratched area were counted.

**Invasion assay**

In brief, A549 and MDA-MB-231 cells seeded at a density of 2 × 10^5^ cells/well in 6-well plate were treated with 25 μM concentration of BBM or equivalent concentration of BBM-NPs for 24 hrs. After that, the cells were trypsinized, collected and 1 × 10^5^ live cells were taken in serum free media and seeded into the matrigel (1:1 ratio of matrigel:serum free media) coated transwell inserts. In the lower chamber of the inserts, DMEM media with serum was added and incubated for 24 hrs at 37 °C. Thereafter, the supernatants were collected from the upper chamber to check the matrix metalloproteinase-2/9 (MMP-2/MMP-9) and vascular endothelial growth factor (VEGF) secreted by the cells to invade through the matrigel. After removing the cells on the upper side of the insert with wet cotton swabs, the cells of lower surface were fixed with 4 % formaldehyde and stained with 0.5 % crystal violet. The photographs of transmigrated cells were taken using inverted phase contrast microscope (Leica QWin, UK) with 10X objective to count the cells those have invaded through the matrigel.

**MMP-2/MMP-9 and VEGF level in the supernatants obtained during invasion assay**

The supernatants obtained from the upper side of the transwell chamber in the aforementioned serum-free invasion assay were analyzed for MMP-2/MMP-9 and VEGF activation. To check the MMP-2/MMP-9 activation, gelatin zymography was performed by following the protocol of Wang *et al* ^5^. The experiment was repeated three times and representative image has been provided. Further the levels of MMP-2 and VEGF in the above supernatants were checked through ELISA Kit [MMP-2 ELISA Kit (Millipore, CA) and VEGF ELISA Kit (Invitrogen, CA)] according to the manufacture’s protocol. The experiment is done twice to check MMP-2 level and result of an independent experiment has been provided. VEGF expression study was done in triplicates and data represented as mean ± SEM.

**Mitochondrial function study**

In brief, A549 and MDA-MB-231 cells seeded at a density 2 × 10^5^ cells in 6-well plate (Corning, NY) were treated with 25 μM BBM or equivalent concentration of BBM-NPs for 48 hrs. Following incubation period, the cells were collected and washed thrice with PBS and incubated with Rhodamine 123 (2.5 μM) at 37 °C for 10 min in the dark. After that, the cells were washed with PBS and taken for analysis using flow cytometer (FACSAria^TM^ Cell sorter, BD Biosciences, CA). The experiment was done in triplicates and representative picture has been provided. Data represented as mean ± SEM.

**Apoptosis study by Flow Cytometry**

In brief, cells seeded at a density of 2 × 10^5^ cells/well in 6-well plate (Corning, NY) were treated with 25 μM BBM or equivalent concentration of BBM-NPs and incubated for 48 hrs. The cells treated with media was taken as control. After 48 hrs, the cells were collected and washed thrice with PBS. After that, the pelleted cells were processed using Annexin V-FITC and PI following the protocol of FITC Annexin V Apoptosis Detection kit (BD Biosciences Pharmingen, CA), and the samples were analyzed by FACScan flow cytometer (FACSCalibar; Becton-Dickinson, San Jose, CA) and FlowJo 4.5 software. The experiment was performed in triplicates and a representative image has been provided. Data represented as mean ± SEM.

**References**

1. Parhi, P. & Sahoo, S. K. Trastuzumab guided nanotheranostics: A lipid based multifunctional nanoformulation for targeted drug delivery and imaging in breast cancer therapy. *J Colloid Interface Sci* 451, 198-211 (2015).

2. Panyam, J., Sahoo, S. K., Prabha, S., Bargar, T. & Labhasetwar, V. Fluorescence and electron microscopy probes for cellular and tissue uptake of poly(D,L-lactide-co-glycolide) nanoparticles. *Int J Pharm* 262, 1-11 (2003).

3. Das, M., Duan, W. & Sahoo, S. K. Multifunctional nanoparticle-EpCAM aptamer bioconjugates: a paradigm for targeted drug delivery and imaging in cancer therapy. *Nanomedicine* 11, 379-389 (2015).

4. Jain, T. K. *et al.* Magnetic nanoparticles with dual functional properties: drug delivery and magnetic resonance imaging. *Biomaterials* 29, 4012-4021 (2008).

5. Wang, S. *et al.* Suppression of growth, migration and invasion of highly-metastatic human breast cancer cells by berbamine and its molecular mechanisms of action. *Mol Cancer* 8, 81 (2009).
